# gpps: an ILP-based approach for inferring cancer progression with mutation losses from single cell data

**DOI:** 10.1186/s12859-020-03736-7

**Published:** 2020-12-09

**Authors:** Simone Ciccolella, Mauricio Soto Gomez, Murray D. Patterson, Gianluca Della Vedova, Iman Hajirasouliha, Paola Bonizzoni

**Affiliations:** 1 Department of Informatics, Systems, and Communication, University of Milano - Bicocca, Milan, Italy; 2 Institute for Computational Biomedicine, Weill Cornell Medicine, New York City, NY USA; 3 Georgia State University, Atlanta, GA USA; 4 Department of Physiology and Biophysics, Weill Cornell Medicine of Cornell University, NewYork City, 10021 NY USA

**Keywords:** Integer linear programming, Hill climbing, Phylogeny, Single cell sequencing

## Abstract

**Background:**

Cancer progression reconstruction is an important development stemming from the phylogenetics field. In this context, the reconstruction of the phylogeny representing the evolutionary history presents some peculiar aspects that depend on the technology used to obtain the data to analyze: Single Cell DNA Sequencing data have great specificity, but are affected by moderate false negative and missing value rates. Moreover, there has been some recent evidence of back mutations in cancer: this phenomenon is currently widely ignored.

**Results:**

We present a new tool, gpps, that reconstructs a tumor phylogeny from Single Cell Sequencing data, allowing each mutation to be lost at most a fixed number of times. The General Parsimony Phylogeny from Single cell (gpps) tool is open source and available at https://github.com/AlgoLab/gpps.

**Conclusions:**

gpps provides new insights to the analysis of intra-tumor heterogeneity by proposing a new progression model to the field of cancer phylogeny reconstruction on Single Cell data.

## Background

Phylogenetics is the field which studies how to reconstruct the evolutionary histories of species, and it has a rich literature [1]. However, phylogenetics has focused on inferring histories from data coming from extant species or individuals, under the assumption that data for ancestor species/individuals are impossible or difficult to obtain.

This is an important difference from cancer progression reconstruction, as in this case, we usually have data from all possible species (or better, from the conceptual analogs of species, that is clones). The clonal theory of cancer [2] postulates that a cancer consists of several clones, that is families of cells carrying the same mutations, that are subject to selective pressure resulting in clonal expansions. In this case, clones play the same role as species in classical phylogenetics.

The easiest way to obtain cancer data from a patient is via a biopsy, where samples from a tumor are extracted, typically using bulk DNA sequencing. This procedure is fairly cheap, but the samples obtained are not very specific: the cells in a bulk-sequencing sample usually belong to several clones. Moreover, we do not know the composition of a sample in terms of clones. Still, by aligning reads extracted from a sample we can obtain (approximately), for each mutation, the fraction of cells in a sample carrying such mutation. Recently, many computational approaches have been developed for the analysis of bulk-sequencing data with the purpose of inferring tumoral subclonal decomposition and reconstructing tumor phylogenies (trees) [3–12], but almost all of them model a tumor progression as the accumulation of mutations under the Infinite Sites Assumption, that is recurrent mutations and mutation losses are not allowed. Notice that, since the coverage of the reads is not perfectly uniform, the fractions that we obtain are only an approximation of the true value. At the same time, given a sufficiently large coverage, the error is small, and the procedure to obtain the data is standard and quite cheap.

An alternative technique is Single Cell DNA Sequencing (SCS): in this case for each cell examined we are able to get the set of mutations it carries. However, this technique is currently expensive and not very reliable, since it produces datasets with a high amount of noise that include allelic dropout (false negatives) and missing values, due to lack of read coverage, as well as false positive calls — although this event is much rarer. Another source of noise is due to doublets, that is signals originating from two separate cells which are erroneously inferred to originate from a single cell: we point out this latter problem is fading away and can be tackled computationally. Still, we need efficient methods that are able to cope with the kind of data that SCS techniques are currently producing, by taming the difficulties due to the noise in data.

Various methods have been developed for this purpose [13–15], some of them introducing a hybrid approach of combining both SCS and VAF data [16–19]. As stated before, most of these methods rely on the Infinite Sites Assumption (ISA) [20], which states that a mutation is acquired at most once in the phylogeny and is never lost. This simplifying assumption also leads to a computationally tractable model of evolution called the perfect phylogeny [21]. However, some recent studies focused on cancer data are finding hints suggesting that the ISA does not always hold [22–24], and so we may need to abandon the strict ISA in this setting. In [23], the authors find that large deletions on several branches of a tree can span a shared locus, thus a given mutation may be deleted independently multiple times. In [24], the authors show that in certain cases, homozygous deletions in cancer genomes can even provide a selective growth advantage. Each (independent) deletion of an acquired mutation takes us further away from the ISA. Some recent methods such as TRaIT [16] and SiFit [15] allow deletions of mutations.

The Dollo model [25] of evolution is designed exactly for some of the cases where a perfect phylogeny does not represent the actual data. More precisely, the Dollo model requires each mutation to be acquired exactly once in the entire history analyzed, while removing all restrictions on the number of times that a mutation can be lost. The Dollo model as well as the Dollo(*k*) variants, where each mutation can be lost at most *k* times, has been introduced recently in the literature on algorithmic approaches for tumor progression inference [12, 26]. Since finding a perfect phylogeny on a complete binary matrix can be solved in linear time [21], several tools have incorporated this model to reduce the running time [27] — but single cell data present a large portion of missing data, which makes the problem much harder.

When the input is an incomplete matrix *M*, the problem of determining if there exists a directed rooted phylogeny *T* obeying the Dollo(1) model and explaining *M* is NP-complete [28] — the proof in that paper is actually on a restriction of directed perfect phylogeny on generalized characters, but it is immediate to notice that those generalized characters correspond to finding a directed Dollo(1) phylogeny from an incomplete binary matrix. On the other hand, when considering standard binary characters, allowing only character gains, and exactly one gain for each character, the problem of finding a directed perfect phylogeny can solved in polynomial time even for incomplete matrices [29].

Moreover, we focus on the Dollo model, which is more general and more computationally expensive than perfect phylogeny model (Dollo(0) is perfect phylogeny), hence requiring even more sophisticated algorithms.

In this paper we propose gpps, an approach which combines Integer Linear Programming (ILP) with a Hill Climbing approach to infer a tumor progression that can include a limited amount of mutation losses, from single cell DNA sequencing data. A contemporary and independent approach for the problem is presented in [30] where the authors propose an ILP formulation and a cutting plane strategy to resolve the problem.

## Results

### Results on real cancer data

We tested gpps on ER ^+^ breast cancer data from [31], consisting of 40 somatic mutations over 47 cells with an estimated false negative rate of 9.73%, a false positive rate of 1.24×10^−6^ and a missing rate of 13.83% (based on the known error rates of the SCS technologies from which this data was obtained); on JAK2-Negative Myeloproliferative Neoplasm data from [32], consisting of 18 mutations over 58 cells, with an estimated false negative rate of 7.63%, a false positive rate of 2.02×10^−5^ and missing rate of 44.82%. Finally we tested gpps on childhood acute lymphoblastic leukemia data of Patient 4 from [33], consisting of 77 somatic mutations across 142 cells, with an estimated false negative rate less than 30% and a zero missing rate. Since the trees proposed in the sequencing papers are manually curated and of high quality, we consider it a good benchmark.

**Fig. 1:**
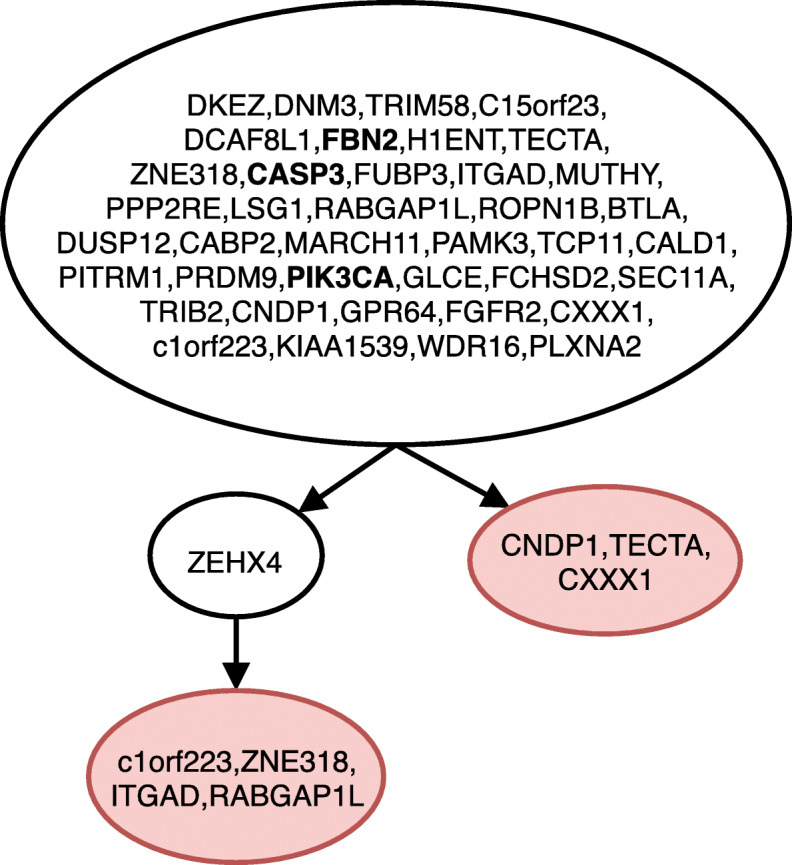
Tree inferred by gpps for ER ^+^ breast cancer patient from [31]. The red-colored nodes indicate deletions of mutations, while mutations highlighted in bold are the mutations indicated as driver in the original sequencing study. Linear paths in the tree have been collapsed for space constraints

**Fig. 2:**
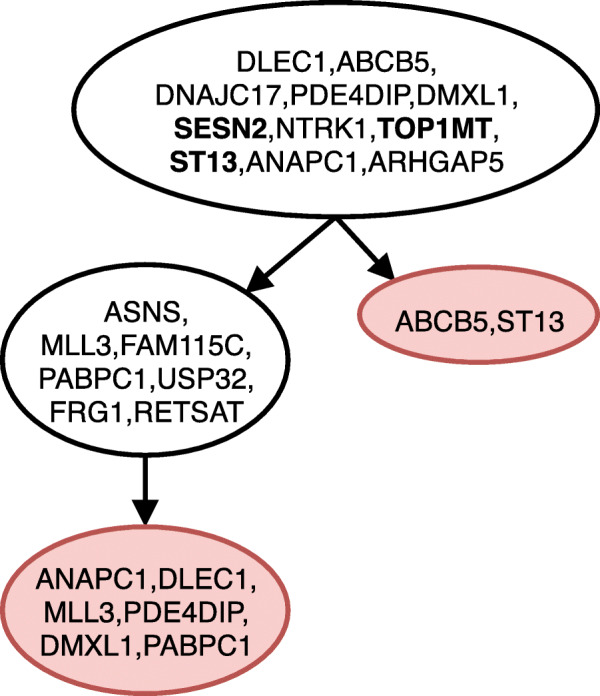
Tree inferred by gpps for JAK2-Negative Myeloproliferative Neoplasm patient from [32]. The red-colored nodes indicate deletions of mutations, while mutations highlighted in bold are the mutations indicated as driver in the original sequencing study. Linear paths in the tree have been collapsed for space constraints

**Fig. 3:**
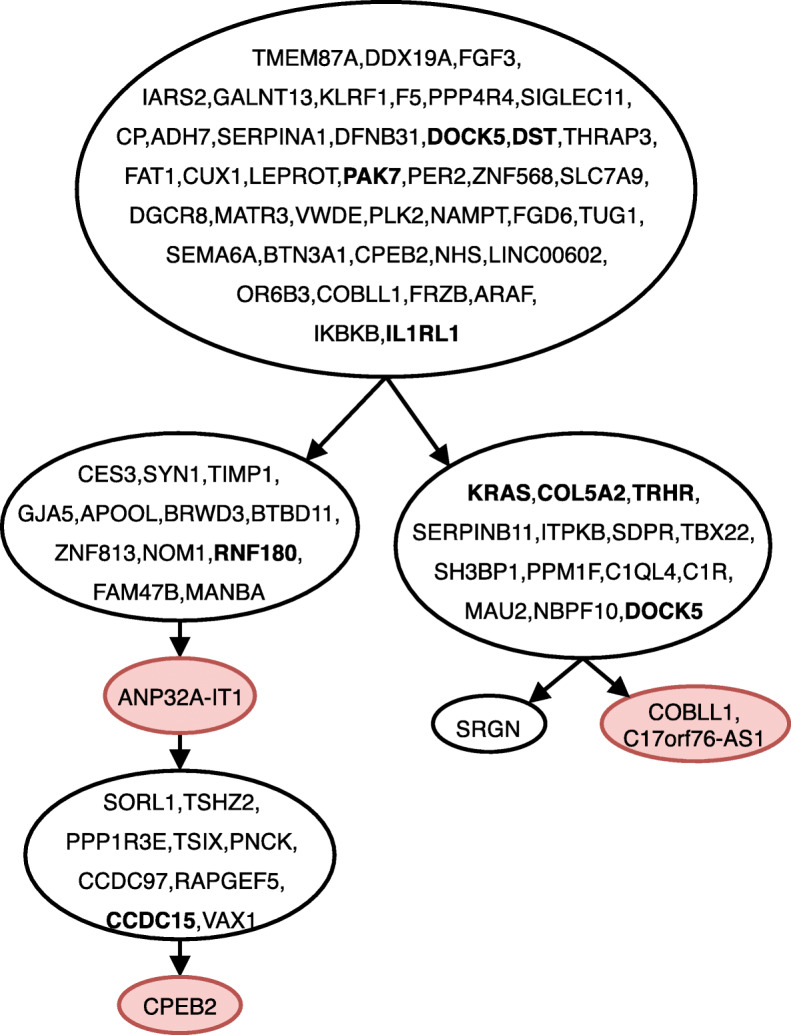
Tree inferred by gpps for childhood acute lymphoblastic leukemia patient 4 from [33]. The red-colored nodes indicate deletions of mutations, while mutations highlighted in bold are the mutations indicated as driver or clonal in the original sequencing study. Linear paths in the tree have been collapsed for space constraints

Figure 1 shows the tree inferred by gpps for ER ^+^ breast cancer patient, where the tree structure assumed in the study is correctly inferred as well as the placement of the driver mutations FBN2, CAPS3 and PIK3C; gpps detected 7 losses, all placed as leaves. Figure 2 shows the tree inferred for a JAK2-Negative Myeloproliferative Neoplasm patient, where similarly to the previous dataset gpps correctly infers the clonal structure and correctly places the driver mutations SESN2, TOP1MT and ST13. As in the previous case it infers 8 losses as leaves. Finally, Fig. 3 shows the tree inferred for childhood acute lymphoblastic leukemia. Once again gpps correctly infers the clonal history proposed in the sequecing study; furthermore it correctly infers the placement of the driver mutations of the four subclonal populations, highlighted in bold in the figure. It also infers 4 mutation losses of which 3 are placed as leaves and one, ANP32A-IT1, is placed as intermediary step in two different subclones.

The solutions were found assuming a Dollo(1) phylogeny model. Furthermore, in the last dataset we forced the solution to have at most 5 losses, while no such restriction was applied to the other two.

### Results on simulated data and comparison with other approaches

We have tested our method on simulated data, where the ground truth is known. We recall that it is possible, however, that a completely different tree achieves a better likelihood than the one obtained via simulation. This problem is essentially unavoidable, since generating a progression that is the unique solution for the corresponding SCS input matrix requires adding artifacts to both the tree and the matrix. It is unlikely that the resulting instance would satisfy even the basic assumptions on cancer progression.

#### Generation of simulated data

Given a fixed number of subclones *S* we generated a random tree of *S* nodes by adding a new node as a child of a random pre-existing one. Each of the *M* mutations $q_{1}, \dots, q_{M}$ is then, uniformly at random, assigned to one of the *s*_*i*_ subclones. We allowed at most a fixed number *k* of deletions in each clonal tree: therefore *k* new nodes are added to the tree at random positions, following the procedure of [19, 27]. A deletion of a mutation is then assigned to each of the *k* new nodes, by picking uniformly at random, one of the mutations which is affecting the parent of the node and which has not been already chosen as a deletion.

To obtain the genotype profile of the *n* cells, we randomly assigned each cell to a node and derived its profile from the clonal tree (independently and uniformly with repetition). Finally, to simulate noise in the data, we flipped a 0 entry to 1 with probability *β* to simulate false positives and a 1 entry to 0 with probability *α* to simulate false negatives. Moreover, each entry has a probability *γ* to be a missing entry. All errors and missing values are uniformly and independently distributed, without repetitions. We simulated a dataset where the number of subclones was fixed to 9, the number of cells and mutations to 100 and 30 respectively; such ratio of mutations over cells is similar to the ones used in recent studies [19, 30]. Lastly, false negative, false positive and missing value rates were 0.1, 10^−4^ and 0.1. These three values where chosen based on the known error rates of the SCS technology we are simulating. For each simulation, at most 5 mutations could be lost, while gpps was run with a Dollo(1) model.

#### Evaluation on simulated data

We measured the accuracy of gpps with two standard cancer progression measures used in various studies [13, 17], defined as follows:

- **Ancestor-Descendant accuracy**: This measure considers all pairs of mutations (*x*,*y*) that are in an ancestor-descendant relationship in the ground truth tree *T*. For each such pair we check whether the ancestor-descendant relationship is conserved in the inferred tree *I*. The score is defined by the F-measure of the preserved relationships in *I*.
- **Different-Lineage accuracy**: Similar to the previous measure, it considers all pairs of mutations (*x*,*y*) that are not in an ancestor-descendant relationship, i.e., are in different branches of *T*. The score is given by the F-measure of the preserved relationship in *I*.

Note that none of the previous metrics account for ISA violations. We decided to compare our ILP (alone) and gpps against SCITE [13] and SiFit [15]. OncoNEM [14] was excluded because it infers cell lineage progressions instead of mutational progression, therefore it is not possible to compare our predictions with theirs; furthermore OncoNEM fails to run on datasets as large as the ones used in the simulations. All the tools were fed with the correct values of false positive and false negatives rates.

**Fig. 4:**
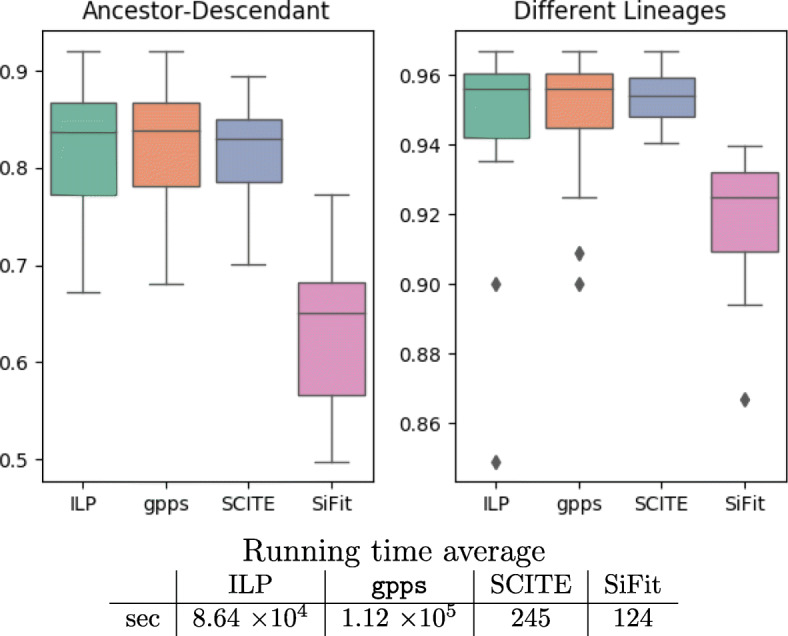
Accuracy results for the simulated data, described in the “Generation of simulated data” section. Our ILP, gpps and SCITE are all relatively close in the Ancestor-Descendant and Different Lineages measures, while SiFit achieves a lower accuracy. It is important to consider that all of these accuracy measures ignore deletions. Finally, we also report runtimes of all tools

**Fig. 5:**
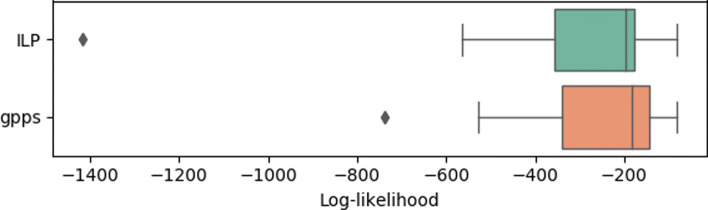
Comparison of the log-likelihood for the ILP and gpps on the simulated data. There is a clear improvement of gpps over the results obtained by the ILP alone, as the likelihood values are overall higher for the whole method

Figure 4 shows the comparison of accuracy between the tools — on average gpps slightly outperforms SCITE in both measures. On the other hand, SiFit achieves a lower accuracy, which is possibly due to the tendency of branching in the model. While HC improves only slightly the mean accuracy of the ILP, it handles outliers, especially in the second measure. Furthermore, as seen in Fig. 5, the HC improves the values of the log-likelihood of the solutions obtained. As already stated, none of the accuracy measures consider the presence of deletions, therefore methods that infer perfect phylogenies are not penalized by these accuracy measures, even if they infer the wrong evolutionary model.

## Discussion

In this paper we have presented gpps: an accurate tool for inferring intra-tumor progression and subclonal composition from SCS data, explicitly incorporating the possibility of mutation losses. The need for models which allow some losses has been established recently [22], and the cases presented there show evidence only for a small number of mutation losses, justifying our focus on the Dollo(1) model.

We have shown that gpps is able to slightly outperform all the other methods available. At the same time, the currently available quality measures are biased against mutation losses, therefore a more complete comparison is necessary before drawing definitive conclusions.

Moreover, we have shown the usefulness of combining a Hill-Climbing step to an ILP approach, since the combination was able to produce better results with fewer deletions, while removing outliers. An additional benefit of this pipeline is that ILP methods might have some issues in scaling to large instances. We have performed some preliminary tests on instances larger than what the current technologies can provide, and those tests did not show any problem. Still, we have incorporated a timeout option in gpps, so that it is able to compute a solution even if the ILP component gets stuck in some solution that is highly suboptimal.

gpps seems to strike a good balance, hence a deeper investigation of this, and related approaches is a worthwhile endeavor.

On real data, gpps performs well and it infers correctly the expected phylogeny tree structures, as well as the driver mutations. In our opinion, the model underlying gpps strikes a good balance, since it is quite simple — there are only two parameters *α* and *β* which are respectively the false positive and false negative rates — while achieving good results. Still, the actual value of the parameters *α* and *β* are usually unknown and affect the overall solution computed by gpps. Therefore it is interesting to study new procedures to infer the best prior values for *α* and *β*.

## Conclusions

There are at least two possible directions for further work that generalize and extend the model and the experimental part. First, we can compare the tools under more general models, such as Dollo(*k*) for larger values of *k* — notice that such an investigation is mainly of theoretical interest, as we have no evidence of such phenomena in nature. Secondly, we can extend the parameter space, for example allowing distinct false positive and false negative rates for each cell and/or mutation. On one hand, it is straightforward to adapt our ILP formulation to this case; on the other hand introducing too many parameters makes the model less informative. Therefore, we need to find a correct tradeoff regarding which new parameters to introduce.

## Methods

In the most abstract formulation, we can see the cancer progression reconstruction problem as a character-based phylogeny reconstruction problem [34] where each character represents the presence/absence of a specific mutation in a cell.

The input to the problem is an incomplete binary matrix *I*, where the entry *I*[*c*,*m*]=0 indicates that the cell *c* does not have the mutation *m*, while *I*[*c*,*m*]=1 indicates that the cell *c* has the mutation *m*. Finally, we denote with *I*[*c*,*m*]= ? where there is not enough information on the presence/absence of mutation *m* in cell *c*. We recall that uncertainty about the presence of a mutation in a cell is a consequence of insufficient coverage in the sequencing, hence it is unavoidable.

However, uncertainty is not the only issue in the sequencing process: the input matrix *I* also contains false positives and false negatives. We assume that these errors occur independently and uniformly across all the (known) entries of *I*. Namely, *E* denotes the predicted matrix, i.e., the binary matrix without missing values computed by the algorithm. In this case, *α* denotes the false negative rate and *β* denotes the false positive rate. In other words, for each pair (*c*,*m*),

- *P*(*I*[*c*,*m*]=0|*E*[*c*,*m*]=0)=1−*β*
- *P*(*I*[*c*,*m*]=1|*E*[*c*,*m*]=0)=*β*
- *P*(*I*[*c*,*m*]=1|*E*[*c*,*m*]=1)=1−*α*
- *P*(*I*[*c*,*m*]=0|*E*[*c*,*m*]=1)=*α*

Our goal is to find a matrix *E* that (1) corresponds to a phylogeny on the set of cells, and (2) maximizes the likelihood

1 $$  P(I|E) = \prod\limits_{c} \prod\limits_{m} P(I[c,m] | E[c,m])  $$

of the observed matrix *I* [13]. In other words, we want to find the phylogeny, as expressed by the matrix *E*, that maximizes the likelihood of the observed matrix *I* [13]. We point out that the values of the unknown entries of the input matrix do not factor into the objective function.

A phylogeny is a rooted labeled tree *T*, where the label set corresponds to the set of mutation gains and losses. The state *S*(*x*) of a leaf *x* in *T* is defined as the set of mutations that are acquired and not lost on the path from the root of *T* to *x*. We say that the tree *T* encodes a matrix *E* if there exists a mapping *σ* of the rows of *E* to the leaves of *T* such that for each row *r* of *E*, it follows that *C*(*r*)=*S*(*σ*(*r*)) where *C*(*r*) is the set of columns which are 1 in *r*, and *σ*(*r*) denotes the leaf of *T* associated with *r* through *σ*. In other words, in the tree *T* we assume that the cell *c* has been extracted from the subpopulation *σ*(*c*).

We can express the likelihood of the matrix *E* as in Eq. 1 — since the involved probabilities are in [0,1] it is convenient to move to a (linear) log-likelihood maximization objective function of the form:

2 $$  \text{max} \sum\limits_{c} \sum\limits_{m} \log P\left(I[c,m] | E[c,m]\right)  $$

### The model of evolution

The Dollo parsimony rule can be interpreted as the impossibility of having an identical mutation in the evolutionary trajectory. This rule can be translated in the phylogeny tree model as the unique introduction of any single mutation but any number of deletions of this mutation.

**Fig. 6:**
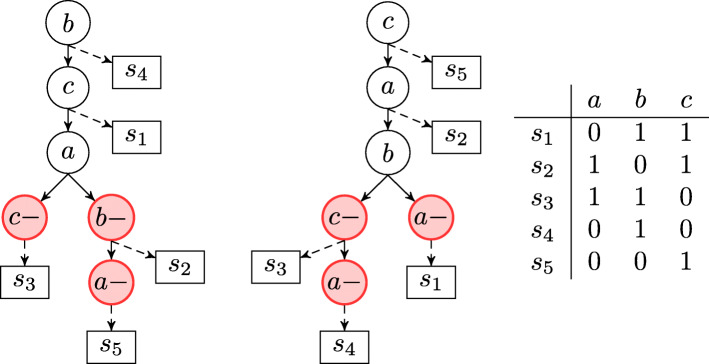
Example of two Dollo phylogenies that explain the same binary matrix. It is important to notice that the ancestral order of mutations *c*,*a* and *b* is inverted but the two different trees can equally explain the input binary matrix. In fact, in a Dollo phylogeny the order of two mutations can be inverted and, due to the introduction of deletions, they could both be correct solutions for a given input

From an algorithmic point of view, phylogeny reconstruction with a Dollo evolutionary model is an NP-complete problem [35, 36]. A hierarchical chain of restricted versions of the model can be obtained by bounding the number of deletions for each character. We denote as Dollo(*k*) the evolutionary model in which each mutation can be acquired exactly once and can be lost at most *k* times. In this way Dollo(0) and Dollo(1) correspond to the perfect [21] and persistent [37–39] phylogeny models, respectively. In the tree generation process for the Dollo(*k*) model (*k*>0) we are required to augment a perfect phylogeny representing the cancer progression by adding nodes which represent the loss of a mutation, i.e., a node labeled $m^{-}_{l}$, representing the potential losses. Observe that losses can appear at any of the *k* copies *m*_*i*_, with 1≤*i*≤*k*, of *m* and that the ordering of the losses is not relevant. The state of the leaf *x* is the set of mutations *m* that, on the path from the root to *x*, have been acquired — the path has a vertex labeled *m*^+^ — but never lost — the path has no vertex labeled $m_{i}^{-}$. We stress that, when deletions are introduced, the set of feasible phylogenies which represent a given solution is no longer unique as in the case of perfect phylogeny — see Fig. 6 for an example.

An exact fixed-parameter algorithm, where the parameter is the number of characters, for testing whether a binary matrix is a persistent model, i.e. a Dollo(1) model has been proposed in [37], while some polynomial time solvable restrictions have been studied in [40]. Testing if a binary matrix *I* has a phylogeny under the Dollo(*k*) model has been attacked via ILP for *k*=1 [41] and for general *k* [12]. Observe that the ILP in [41] is based on a previous work on completing missing entries of a binary matrix via ILP to get a perfect phylogeny [42]. We will exploit the latter formulation in [12], as well as its extension to incomplete matrices [43], to describe an ILP approach for tumor phylogeny reconstruction from single cell data.

We will exploit the latter formulation to describe an ILP approach for tumor phylogeny reconstruction from single cell data.

First, we recall that a well known characterization of perfect phylogenies states that a *complete* binary matrix *M* has a directed perfect phylogeny if and only if it has no *conflicting* pair of columns — two columns are in conflict if they contain all three configurations (0,1), (1,0),(1,1) — inducing the so-called forbidden matrix [21].

The ILP formulation on *incomplete* matrices [42] essentially consists of introducing a binary variable for each missing entry, and describing a set of constraints towards the goal of minimizing the conflicting pairs.

**Fig. 7:**
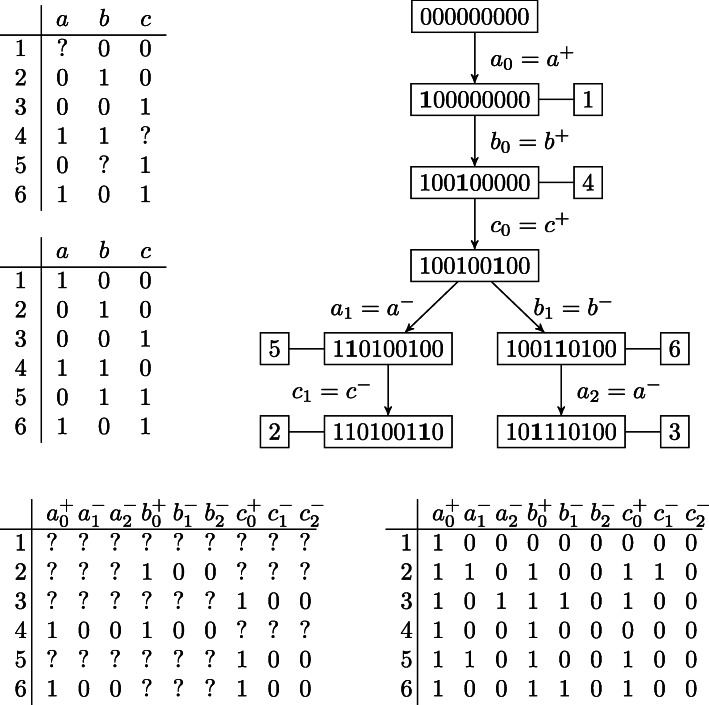
An input matrix *M* (top left), a Dollo(2) completion *M*^∗^ (center left) and its corresponding phylogeny tree *T* (top right). The corresponding extended matrix *M*_*e*_ (bottom left) and a completion $M_{e}^{*}$ (bottom right) according to Proposition 1. In the tree, boldfaced characters correspond to changes between each node and its parent

To adapt this approach to persistent phylogenies [41] to our setting — Dollo(*k*), we need a property (see Fig. 7 for an illustration):

#### **Proposition 1**

[12] Let *M* be an incomplete binary matrix. Let *M*_*e*_ be the (incomplete) extended binary matrix obtained from *M* as follows: for each entry *M*[*i*,*j*] we have the entry *M*_*e*_[*i*,*j*^+^] and *k* entries $M_{e}\left [i,j_{l}^{-}\right ]$ (for 1≤*l*≤*k*) such that (1) if *M*[*i*,*j*]=1 then *M*_*e*_[*i*,*j*^+^]=1 and $M_{e}\left [i,j_{l}^{-}\right ] = 0$ for 1≤*l*≤*k*, (2) if *M*[*i*,*j*]=0 or *M*[*i*,*j*] is missing, then the entries $M_{e}\left [i,j^{+}\right ], M_{e}\left [i,j_{l}^{-}\right ]$ are all missing. Then *M* has a completion *M*^∗^ that has a Dollo (*k*) phylogeny if and only if *M*_*e*_ has a completion $M^{*}_{e}$ that has a perfect phylogeny such that $M^{*}_{e}\left [i,j^{+}\right ] - {\sum \nolimits }_{l\le k} M^{*}_{e}\left [i,j_{l}^{-}\right ]=M\left (i,j\right)$ if *M*(*i*,*j*)∈{0,1} and $M^{*}_{e}\left [i,j^{+}\right ]\ge {\sum \nolimits }_{l\le k} M^{*}_{e}\left [i,j_{l}^{-}\right ]$ if *M*(*i*,*j*) is missing.

Our main contribution is combining the ILP formulation of [12] with the definition of tumor perfect phylogeny reconstruction from single cell data, to obtain a new ILP formulation, and then augmenting it with a Hill Climbing approach, for tumor phylogeny reconstruction from single cell data that incorporates mutation losses in the model.

### The ILP formulation

In this section we present our ILP formulation for the tumor phylogeny reconstruction from single cell data. We recall that the input of the problem is an incomplete matrix *I* represented as a set of binary variables *I*(*c*,*m*) such that *I*(*c*,*m*)=1 if cell *c* has (according to the input data) the mutation *m*, while *I*(*c*,*m*)=0 if cell *c* does not have (according to the input data) the mutation *m*. Notice that the input data is incomplete, hence we can have pairs (*c*,*m*) such that the variable *I*(*c*,*m*) does not exist.

The variables *E*(*c*,*m*^+^) and $E\left (c,m_{i}^{-}\right)$ encode the extended matrix that we want to compute and that will satisfy Proposition 1. Differently from the variable *I*(·,·), for each pair (*c*,*m*), all variables *E*(*c*,*m*^+^) and $E\left (c,m_{i}^{-}\right)$ exist.

We introduce some auxiliary variables that help in making the ILP formulation easier to read. The binary variables *F*(*c*,*m*) indicate if, in the predicted matrix, the cell *c* has the mutation *m*. By Proposition 1, *F*(*c*,*m*)=1 if and only if *E*(*c*,*m*^+^)=1 and all $E\left (c,m_{i}^{-}\right)$ are equal to zero. Moreover, the real variables *w*(*c*,*m*) represent the probability of *E*(*c*,*m*) given *I*(*c*,*m*) — the formula of the actual values depends on the possible cases, that is if we have a true positive, a true negative, a false positive, and a false negative.

To establish if two columns are in conflict, we introduce the final binary variables *B*(*p*,*q*,*a*,*b*), which are defined for each pair of columns (*p*,*q*) and for each possible pair of values (*a*,*b*)∈{(0,1),(1,0),(1,1)}. More precisely, *B*(*p*,*q*,*a*,*b*) indicates if for the pair (*p*,*q*) of columns there exists a cell *c* where *E*(*c*,*p*)=*a* and *E*(*c*,*q*)=*b*. Notice that two columns *p* and *q* are conflicting iff *B*(*p*,*q*,0,1)+*B*(*p*,*q*,1,0)+*B*(*p*,*q*,1,1)=3. We are now ready to introduce our ILP formulation, where we use *C* to denote the set of cells (i.e., the rows of the input matrix *I*), *M* to denote the mutations (i.e., the columns of *I*), and *M*^∗^ to denote the set of possible mutation gains or losses.

Finally, the objective function is the logarithm of the likelihood of the inferred matrix *F* given the input matrix *I* — this allows to express the objective function as a summation, instead of a product. Moreover, notice that Eq. (3) is a sum of log*w*(*c*,*m*) terms, which apparently is not a linear function. But Eqs. (5) and (6) show that *w*(*c*,*m*) is actually a linear function of *F*(*c*,*m*): since *F*(*c*,*m*) is a binary variable that can be only 0 or 1, a trivial manipulation allows us to replace log*w*(*c*,*m*) with a linear function of *F*(*c*,*m*) — such function is omitted for the sake of clarity.

3 $$\begin{array}{*{20}l} \max \sum\limits_{c\in C}\sum\limits_{m\in M}\log w(c,m),\text{ subject to} &  \end{array} $$

4 $$\begin{array}{*{20}l} F(c,m) = E\left(c,m^{+}\right) - \sum\limits_{i\le k} E\left(c, m_{i}^{-}\right) & \ \forall c\in C,\ m\in M \end{array} $$

5 $$\begin{array}{*{20}l} w(c,m) = \left(1 - \alpha\right) F(c,m) + \beta \left(1 - F(c,m) \right)\quad & \text{ if} I(c,m) = 1 \end{array} $$

6 $$\begin{array}{*{20}l} w(c,m) = \alpha F(c,m) + \left(1 - \beta\right) \left(1 - F(c,m) \right)\quad & \text{ if} I(c,m) = 0 \end{array} $$

7 $$\begin{array}{*{20}l} B(p,q,0,1)\ge E(c,q) - E(c,p) \quad & \ \forall c\in C,\ p,q\in M^{*} \end{array} $$

8 $$\begin{array}{*{20}l} B(p,q,1,0)\ge E(c,p) - E(c,q) \quad & \ \forall c\in C,\ p,q\in M^{*} \end{array} $$

9 $$\begin{array}{*{20}l} B(p,q,1,1)\ge E(c,p) + E(c,q) -1 \quad & \ \forall c\in C,\ p,q\in M^{*} \end{array} $$

10 $$\begin{array}{*{20}l} B(p,q,0,1) + B(p,q,1,0) + B(p,q,1,1)\le 2 \quad & \forall p,q\in M^{*} \end{array} $$

$$\begin{array}{*{20}l} B\left(\cdot,\cdot,\cdot,\cdot\right), F\left(\cdot,\cdot\right), E\left(\cdot,\cdot\right) & \in \{0,1\}\notag \end{array} $$

The total number of variables and constraints in the formulation are *O*(*nm*+*m*^2^) and *O*(*nm*^2^) respectively.

Recent methods [44] assume that it is unrealistic to model false positives and false negatives occurrences as independent with a fixed probability for all cells and thus propose using different values for each. While this is not explored in our paper, we notice that it is fairly trivial to extend the above ILP formulation to introduce non-uniform values (which are still given as input): we change constraints (5) and (6) to use *α*_*c*,*m*_ and *β*_*c*,*m*_ instead of *α* and *β*. Since the new values *α*_*c*,*m*_ and *β*_*c*,*m*_ would still be user-given constants the formulation still holds identically.

### Software implementation: gpps

Our approach has been implemented in Python, the resulting program called gpps. The program generates the ILP formulation which is fed to an ILP solver in order to get the optimal solution. In our experiments we have used Gurobi 8.0 as the ILP solver. Additionally, we have introduced a timeout for a run, since the generated ILP problem could be large and its resolution could require a considerable amount of time. We exploit the fact that Gurobi can be halted at any time and it returns the best feasible solution computed so far. Hence, imposing a timeout allows the ILP solver to compute a solution with a small total error.

Since the solution produced by ILP with a timeout will be suboptimal, we used a local search algorithm to continue the exploration of the solution space starting from the output of the ILP. We implemented a variation of the standard *Hill Climbing* (HC) search — which iteratively moves from a starting point to all the surrounding neighbors optimizing a given function. The best scoring neighbor is set as the new starting point and the process continues until there is no new solution that improves the current best one.

**Fig. 8:**
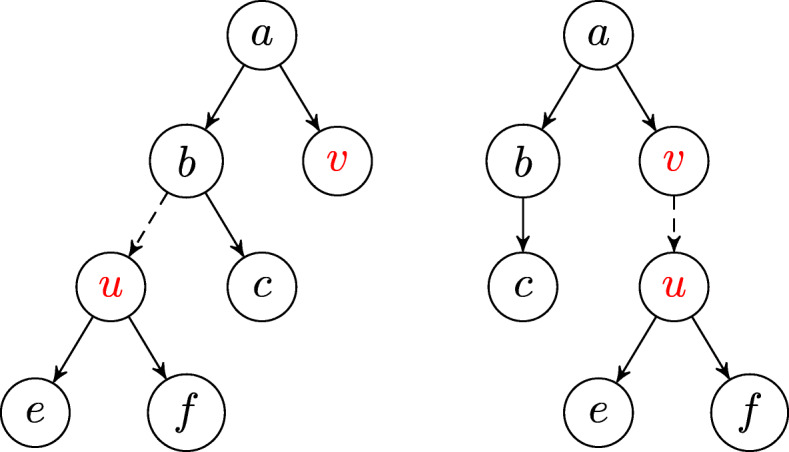
A tree (left) and its neighbor (right) via a Subtree Prune and Reattach operation where we prune the subtree rooted in *u* and reattach it as a child of *v*. Note that this operation is not to be confused with the well-known *Subtree Prune and Regraft* operation [45] for binary leaf-labeled trees

In our case, we say that a tree $\widetilde {T}$ is a *neighbor* of tree *T* if there exist two nodes *u*,*v*∈*T* such that, by pruning the subtree rooted in *u* and by reattaching it as a child of *v*, we obtain the tree $\widetilde {T}$ — such operation is called *Subtree Prune and Reattach*, see Fig. 8. In moving a subtree of *T* to another part of the tree, nodes representing the loss of a mutation may no longer apply. However, in this case, all such nodes can simply be contracted, i.e., by removing the node and adding an edge from its father to its child (which is necessarily unique, if it has one). For example, if *f* in the tree on the left in Fig. 8 is instead the loss *b*^−^ of mutation *b*, this no longer applies in the subtree on the right after the Subtree Prune and Reattach operation, because mutation *b* is no longer acquired above this subtree — we simply remove this node *b*^−^. Note that since loss nodes are only removed, the Dollo(*k*) property of a tree is preserved in performing this operation.

Since, according to the Subtree Prune and Reattach operation, the neighbors of a tree are the set of trees generated by all possible pairs of nodes *u*,*v*∈*T*, the size of this neighborhood is quadratic in the size of *T*, which is not computationally feasible. For this reason we modified the standard HC algorithm by generating *N* random neighbors of the starting point at each iteration, instead of exploring all of the surrounding solutions, and then stopping the algorithm after *M* iterations — where *N* and *M* are user-defined parameters. The final result of this process is thus the best solution explored in this modified HC phase.

## Data Availability

The datasets we used in the current study are publicly available at https://github.com/AlgoLab/gpps.

## Abbreviations

DNA: Deoxyribonucleic acid
VAF: Variant allele frequency
ILP: Integer linear programming
HC: Hill climbing
SCS: Single cell sequencing
ISA: Infinite sites assumption
